# Effectiveness of transcervical hysteroscopic endometrial resection based on the prevention of the recurrence of endometrial polyps in post-menopausal women

**DOI:** 10.1186/s12905-015-0179-0

**Published:** 2015-02-22

**Authors:** Jesus S Jiménez-Lopez, Ana Granado-San Miguel, Alvaro Tejerizo-Garcia, Jose L Muñoz-Gonzalez, Gregorio Lopez-Gonzalez

**Affiliations:** Service of Obstetrics and GynaecologyUniversity Hospital 12 of October, Avenue of Córdoba s/n, 28041 Madrid, Spain

**Keywords:** Endometrial resection, Polyp in menopause, Endometrial polyp recurrence

## Abstract

**Background:**

The objectives of this study were to determine the effectiveness the effectiveness of post-polypectomy hysteroscopic endometrial resection in preventing the recurrence of endometrial polyps in post-menopausal patients and analyse the complications and necessity of additional surgery in patients, in addition to their degree of satisfaction.

**Methods:**

A prospective longitudinal study of post-menopausal patients diagnosed with endometrial polyps was conducted including polypectomy and hysteroscopic endometrial resection following the therapeutic purposes (endometrial polyp removal) and prevention of recurrence of endometrial polyps.

We evaluated the general condition and characteristics of the patients, including age, BMI, smoking habits, medical, surgical, and obstetrics history and menstrual status. The results were analysed at several time points, 6, 18, 42 and 60 months by hysteroscopy, including the presence of vaginal bleeding and/or possible intracavitary pathology.

**Results:**

A total of 89.5% (n = 355) of our patients had profile factors associated with the increased incidence of endometrial polyps and hyperestrogenism (diabetes mellitus, hypertension and overweight); 89.5% (n = 355) of patients were overweight; 34% had grade I obesity. The surgical procedure was safe, with a 90% (n = 357) success rate without complications, which was higher than the 95–99.5% at the beginning and end time points of the study. Patient acceptance and satisfaction was 90 and 84%, respectively.

**Conclusions:**

Endometrial resection proved effective in preventing the recurrence of endometrial polyps. It is a safe and effective method. Post-menopausal bleeding reduces the presence of endometrial polyps. Patients reported satisfaction and acceptance of the procedure.

## Background

Endometrial polyps are a formation of the uterine mucosa (focal proliferation of the basal layer) and usually involve the epithelium and variable amounts of glands and stroma. Polyps are organised on the vascular matrix together with the uterine wall, which provides a more or less ample surface [1].

The frequency of this type of polyp is not known, although it is believed to be approximately 24%. The majority of cases are asymptomatic, and typically, endometrial polyps are accidentally discovered during the generalised use of diagnostic techniques such as transvaginal ultrasound or hysteroscopy. Diagnosis and treatment are increasingly frequent in asymptomatic patients [2-4].

In 80% of the cases, such polyps appear in an isolated form, although there also can be multiple polyps. In post-menopausal patients, the polyp glands are atrophic epithelial tissue and surrounded by fibrous stroma [5].

When symptoms occur, there is typically post-menopausal bleeding. In large polyps protruding from the external cervical os, there may be pain from cervical dilation, and bleeding is usually more abundant.

The definitive diagnosis is pathological, although the suspicion is verified by transvaginal ultrasound, which can even identify the pedicle by colour Doppler. Sonohysterography or hysteroscopy reveals the morphology, and the latter is the diagnostic method of choice because it allows for perfect definition and biopsy decisions [6].

The treatment of choice is the removal of the polyps, and hysteroscopic polypectomy is the “gold standard” but requires trained personnel [7]. The procedure can allow a decision in case of pathologic malignancy, although in low-risk cases, asymptomatic women without risk factors, if they have a single polyp of less than 1 cm in postmenopause, expectation may be sufficient [8-10]. The risk of malignancy was significantly correlated with increasing age and menopausal status, polyp size larger than 1.5 cm and hypertension and tamoxifen usage [11-18].

Some women tend to develop recurrent endometrial polyps, but the actual incidence is unknown. Therefore, there is a need for research on the long-term rate of endometrial pathology in large cohorts of post-menopausal women. Few studies have specifically addressed the management of intrauterine lesions in post-menopausal women despite the fact that endometrial polyps are more common in this age group.

Post-polypectomy recurrence varies depending on the surgeon’s experience, technique, training and characteristics involved. A recurrence rate of 15% has been previously been reported [19]. In another series involving special characteristics in patients (breast cancer and tamoxifen treatment), the recurrence rate was much higher, 29.7% [19-23].

The long-term impact of post-polypectomy endometrial resection as a prophylaxis method for recurrent endometrial polyps in post-menopausal women remains unknown. In the present study, we followed patients up to 60 months post-resection. In the initial procedure, the patients underwent hysteroscopic polypectomy, followed by endometrial resection. This study sets out to determine the effectiveness of polypectomy hysteroscopic endometrial resection in preventing the recurrence of endometrial polyps in post-menopausal patients. We analyse the complications, additional surgeries and related patient satisfaction.

## Methods

**Figure 1:**
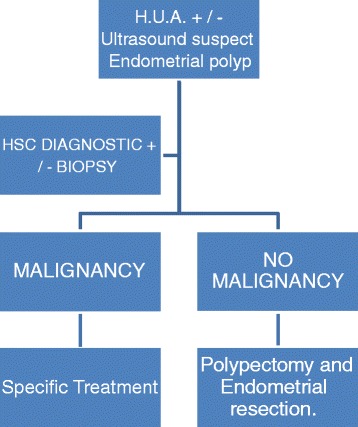
**Algorithm management of endometrial polyps in post-menopausal patients.**

Our study group consisted of patients with post-menopausal bleeding and/or ultrasonographic endometrial thickening that were submitted to hysteroscopy with biopsy. For the group of patients who were diagnosed with post-menopausal endometrial polyps, a prospective longitudinal study was proposed and conducted concerning hysteroscopic polypectomy and hysteroscopic endometrial resection following the therapeutic purposes (endometrial polyp removal) and the prevention of endometrial polyp recurrence. After the procedure and follow-up time periods were explained, all patients signed informed consent forms. The study was approved by the local Ethics Committee of 12 Octubre Hospital. Madrid . Spain (11/2006).All women experienced vaginal bleeding and/or displayed sonographic suspicion of this disease, in addition to being studied in the Endometrial Pathology Unit, Department of Obstetrics and Gynaecology, Hospital 12 de Octubre in Madrid between 2006–2012. We evaluated the following general characteristics of the patients: age; BMI; smoking habits, medical and surgical history; obstetric status and menstrual status. Subsequently, individual gynaecological ultrasonography was performed along with diagnostic hysteroscopy with endometrial biopsy. After performing polypectomy and endometrial resection with a conventional resectoscope, we analysed the reported results of the follow-ups performed at 6, 18, 42 and 60 months in which hysteroscopy was assessed by the presence of secondary treatment complications: vaginal bleeding and/or possible intracavitary pathology (Figure 1).

Our study group is composed of a total of 397 post-menopausal patients, including symptomatic post-menopausal (abnormal uterine bleeding) patients (n = 161), with or without ultrasound suspected endomyometrial pathology and ultrasound findings of asymptomatic patients with suspected endometrial polyps (n = 236).

In all patients, cervical and endometrial malignancy was ruled out before the intervention.

Treatment was considered successful if there was an absence of pathology detected during intracavitary hysteroscopy performed after surgery. Just as the absence of endometrial polyp in subsequent revisions of study time.

Resection and/or endometrial ablation was performed in the operating room with the patient in a lithotomy position and under local anaesthesia, regional or general depending on the patient characteristics. A rigid 250 × 4 mm optical resectoscope was used with an outer sheath that allowed the return of the expansion medium and an internal one for irrigation and resection cavity with cutting and coagulation electrode. Monopolar energy was transmitted through a 100 w intensity electric generator (power cut). The continuous flow of expansion medium (5% glycine) was regulated by an automatic irrigation pump.

To assess the degree of patient satisfaction with treatment and acceptance thereof, patients were telephoned at random. The surveyor was blinded to the patient’s treatments characteristics. The survey consisted of three direct questions with three possible answers: yes, no and do not know/no answer.

- Are you satisfied with the treatment received?
- Was the treatment received acceptable?
- Would you recommend this treatment to another person?

Statistical analysis was performed using SPSS 15.0. In all cases, we used the bilateral contrast hypothesis for the calculation of p, taking as statistically significant values less than 0.05.

To determine the relationship between two quantitative variables, the mean comparison procedure was performed with the *T*-TEST GROUPS SPSS system, which calculates the value of Student’s *t* test. When the variables did not follow a normal distribution according to the Kolmogorov-Smirnov test, the analysis was performed using the nonparametric Mann–Whitney test.

The qualitative relationship between categorical variables was performed with the SPSS system CROSSTAB procedure, which is equivalent to a logistic regression model containing a simple one-predictor variable. Yates correction was applied to the chi-square test when the frequency of the observations in any cell was less than 10. When the expected frequencies were less than 5, Fisher’s exact test was used for the analysis.

## Results

The mean age of the patients was 61.25 years (SD 0.71), 58.43 years (SD 0.99) in patients who had post-menopausal bleeding and 62.9 years (SD 1.15) for asymptomatic patients with clinical suspicion of pathological intracavitary ultrasound findings.

In regards to the medical conditions that are associated with increased incidence of endometrial polyps and hyperestrogenism (diabetes mellitus, hypertension and overweight), 89.5% (n = 355) of our patients had these features.

A total of 89.5% (n = 355) of patients were overweight, a prevalence well above the population mean. When analysing the results by degrees of obesity, most of these patients were overweight (35%) or presented grade I obesity (34%) (Table 1).

**Table 1:** **BMI postmenopausal women**

**BMI**	**%**	**n**	
BMI < 20	1%	n = 4	
BMI 26–30: overweight	35%	n = 139	GLOBAL OVERWEIGHT
BMI 31–35: OBESITY I	34%	n = 135	**n = 355**
BMI 36–40: OBESITY II	11,5%	n = 46	**89,5%**
BMI > 40: OBESITY III	9,5%	n = 38	

Body Mass Index.

A total of 95% (n = 377) of patients did not smoke and thus were not influenced by the hypoestrogenism created by this toxic habit. The mean age of menarche was 12.96 years (SD 0.2), and the mean age of menopause was 50 years (SD 0.45).

Of the post-menopausal patients studied, 30% (n = 119) had a post-menopausal abnormal uterine bleeding, 9.5% (n = 38) had abnormal uterine bleeding associated with a suspected pathology on intracavitary ultrasound and 60.5% (n = 240) had ultrasound findings.

In our population, 31% (n = 123) of cases had taken tamoxifen for an average duration of 64.83 months (SD 9.02), and hormone replacement therapy was received by 17.5% (n = 69) patients for an average of 78.13 months (SD 19.18).

The surgical procedure used was safe, and 90% (n = 357) of the population suffered no complications in the short to medium term. However, 2% (n = 8) of patients suffered cervical laceration during surgery; another 2% (n = 8) had uterine perforation, which was treated conservatively; and another 2% (n = 8) suffered fluids overloads without electrolyte disturbances. Other complications included infection (0.25%, n = 1), such as acute endometritis, transient haemorrhage (0.25%, n = 1) and post-puncture headache (3%, n = 12). In the study group, there were four hysterectomies (1.0%). There were two during the follow-up diagnosis because of endometrial adenocarcinoma (0.5%), one for pelvic floor pathology (uterine prolapse) and the presence of an endometrial polyp with proliferative activity, and the fourth was for ovarian neoplasm. The success rate was 99.5, 97, 95.7 and 95%, respectively, for time points 6, 18, 42 and 60 months post-resection (Table 2).

**Table 2:** **Overall efficacy in patients postmenopausal endometrial resection and prevention of recurrence of endometrial polyp**

**6 Months**	**18 Months**	**42 Months**	**60 Months**
**Success**:	**Success**:	**Success**:	**Success**:
99.5% (n = 395)	97% (n = 385)	95.7% (n = 380)	95% (n = 377)
**Failure**:	**Failure**:	**Failure**:	**Failure**:
0.5% (n = 2)	3% (n = 12)	4.3% (n = 17)	5% (n = 20)
			**p < 0.00001**

Global Effectiveness of Resection in postmenopausal ablation.

In analysis of the success based on certain factors such as tamoxifen treatment, the success rate was 93, 90, 88 and 87%, respectively.

The percentage of patients that considered the treatment acceptable and were satisfied were 90 and 84%, respectively. Similarly, 70% of patients would recommend this treatment (Table 3).

**Table 3:** **Level of satisfaction and acceptability of endometrial resection in postmenopausal patients**

**Satisfaction and treatment acceptability satisfaction**	YES: 87% (n = 345)
	NO: 13% (n = 52)
**Acceptability**	YES: 80,5% (n = 320)
	NO: 17,5% (n = 69)
	NS/NC: 2% (n = 8)
**Recommendation to another patient**	YES: 67,5% (n = 268)
	NO: 13% (n = 52)
	NS/NC: 19,5% (n = 77)

(Yes. The patient considers proper treatment and is pleased with the results. I recommend it to other patients.

No. The patient does not consider the appropriate treatment and not satisfied with the results. And is not recommended to other patients.

N/C. The patient does not have a strong opinion).

## Discussion

Endometrial polyps are a common condition that affects peri- and post-menopausal women more frequently. It is usually associated with abnormal uterine bleeding, although it may be asymptomatic. In post - menopausal patients, these polyps require special attention, as the risk of malignancy is variable (0.8 to 4.8%) [24], and histopathologic examination, preferably guided biopsy with hysteroscopy, is necessary. The second point that requires special attention is the recurrence of endometrial polyps. Studies of recurrent endometrial polyps after hysteroscopic polypectomy after up to nine years of follow-up report rates of 2.5% to 3.7% [25,26]. The recurrence of endometrial polyps is one of the most common problems in breast cancer patients receiving long-term treatment with tamoxifen. Each year of additional use of tamoxifen is estimated to increase the risk of developing recurrent polyps by up to five-fold, and 7.7% of tamoxifen-treated post-menopausal patients may develop recurrent endometrial polyps. Gao et al. [27] found that patients treated with tamoxifen that underwent polypectomy associated with endometrial resection had an endometrial recurrence rate of 8.54%. In response to the question of whether a strategy would be effective in these circumstances, we hypothesised that to prevent recurrence of this condition, a simultaneous endometrial polypectomy with endometrial resection should be performed. Polypectomy with endometrial ablation could reduce abnormal vaginal bleeding and pathological sonographic findings, which therefore would reduce the need for or completely avoid the need for invasive procedures, such as hysteroscopy or curettage. This would, in turn, avoid the need for the use of anaesthetic and surgical resources. A mid- to long-term study can inform us if this minimally invasive alternative to other strategies can prevent the recurrence of post-menopausal endometrial polyps and provide endometrial protection in association with the long-term use of tamoxifen.

In our series, most patients were asymptomatic, with the diagnosis of endometrial polyps coming from suspected incidental findings in ultrasonography 60.5% (n = 62). The remaining 39.5% of patients (n = 42) also displayed clinical symptoms (post-menopausal genital bleeding). These figures are in between the range found by other authors (7.4 - 15.6% and 53.9%).

Despite the exclusion of endomyometrial malignancy cases, these patients had risk factors for endometrial polyp recurrence and, of course, for adenocarcinoma of the endometrium [hyperestrogenism with a high rate of obesity (89.5%), 31% with tamoxifen treatment for at least five years, and most of the patients were smokers]. The study group patients did not report early menarche or late menopause.

The use of endometrial resection in our study group was verified as an effective preventive measure against endometrial polyp recurrence in post-menopausal patients. The incidence of relapse in our patients significantly decreased with time. We observed a decrease in the recurrence from the third follow-up time point (18 months post-resection; Table 2).

Experience in the use of preventive endometrial resection and endometrial polyp recurrence is limited, but some reports indicate the decline in the incidence of recurrent polyps in the patients studied [23,27,28].

We considered treatment success to be defined by the absence of endometrial polyp recurrence according to diagnostic hysteroscopy. The success rates in the study population were 99.5, 97, 95.7 and 95%, respectively, depending on the follow-up time point (Table 2). If the analysis is performed considering possible factors that favour the recurrence of polyps, such as treatment with tamoxifen, the success rate for preventive endometrial polyp recurrence is similar to the general population. In the first year of follow-up with normal hysteroscopy, Capuano et al. [28] reported an 89% success rate. These results could be explained by the destruction of the lining of the endometrium with the consequent impossibility of endometrial polyp recurrence despite the existence of oestrogen stimulation.

The safety of the treatment, in the perimenopausal period is very high. In fact, 90% of patients did not experience any side effects in the short to medium term, and the remaining 10% suffered only minor complications. However, they were two cases of endometrial adenocarcinoma post-ablation, which can be explained by the persistence of endometrial tissue after resection. This complication is very important and highlights the careful selection of patients and the obligation to verify the absence of endometrial malignancy prior to performing endometrial resection.

In our study, we performed hysterectomies in 1.0% of cases because of malignancy, with two other cases altering the pelvic floor and one complex adnexal tumour. Similar figures have been reported in other series [27,28]. The probability of avoiding additional surgical interventions was greater than 90% after 60 months of follow-up.

These results confirm previous studies supporting the use of endometrial resection [27,28], especially in selected cases receiving treatment with tamoxifen.

The implementation of minimally invasive surgical treatments is well tolerated and accepted by patients. A total of 87% of our patients expressed satisfaction with the surgical procedure and postoperative care received. The patients reported a high degree of satisfaction and high acceptability.

## Conclusions

Endometrial resection proved to be an effective method in the prevention of endometrial polyp recurrence. Thus, our results demonstrate the usefulness of endometrial resection, especially in selected cases receiving tamoxifen therapy. The method is a safe and effective treatment to decrease post-menopausal bleeding by reducing the presence of endometrial polyps. The procedure was valued by patients who had high levels of satisfaction and acceptance.
